# Supplementary material for: Varied and unexpected changes in the well-being of seniors in the United States amid the COVID-19 pandemic
Source: PLoS One. 2021 Jun 17;16(6):e0252962. doi: 10.1371/journal.pone.0252962 (PMC8211190; doi:10.1371/journal.pone.0252962)
Supplement: S1 File — (DOCX) [file pone.0252962.s001.docx]

**S1 File. Survey Instrument: Wave 1**

Notes to programmer are given in brackets [.]

**INTRO: Thanks for participating in our study! This survey asks you about your health and how you feel about your life.**

**Some of the questions ask you about your past, about what happened and how you felt. Some ask about the recent past like yesterday and others about a more distant past like the past 12 months.**

**To help you, the time period for each question is shown in bold.**

**Q1.**

What is your date of birth? [drop down menu for each]

DD MM YYYY

[If age at time of interview is not between 60 and 68, please thank respondent and terminate survey]

**Q2.**


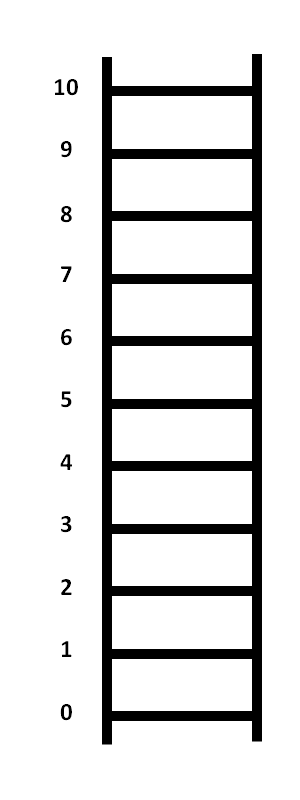
Please imagine a ladder with steps numbered from zero at the bottom to ten at the top. The top of the ladder represents the best possible life for you and the bottom of the ladder represents the worst possible life for you. On which step of the ladder would you say you personally feel you stand at this time?

10 Best possible

09

08

07

06

05

04

03

02

01

00 Worst possible

**Q3.**

In general, how satisfied are you with your life?

1. Very satisfied
2. Somewhat satisfied
3. Neither satisfied nor dissatisfied
4. Somewhat dissatisfied
5. Very dissatisfied

**Q4.**

[Please show one question per screen, randomize the order in which the questions are presented]

Did you experience the following feelings during A LOT OF THE DAY **yesterday**? Yesterday, did you experience _____?

Q4.1. Enjoyment

Q4.2. Physical Pain

Q4.3. Worry

Q4.4.Sadness

Q4.5. Stress

Q4.6. Anger

Q4.7. Happiness

Response scale:

1Yes

2 No

**Q5.**

Q9.1. Did you worry about money **yesterday**?

1 Yes

2 No

**Q6.**

In general, would you say your health is:

1. Excellent
2. Very Good
3. Good
4. Fair
5. Poor

**Q7.**

In general, would you say your ***mental health*** is:

1. Excellent
2. Very Good
3. Good
4. Fair
5. Poor

**Q8.**

The following to questions ask about how you have been feeling in the **past 2 weeks**.

**Over the last 2 weeks**, how often have you been bothered by any of the following problems?

Little interest or pleasure in doing things

1. Nearly every day
2. More than half the days
3. Several days
4. Not at all

**Q9.**

Which of the following words is similar to **love**?

1. Wall
2. Line
3. Cynical
4. Like
5. Keyboard

**INTRO 2: Now we will ask you some questions about your medical care use**

**Q10.**

**In the next month,** are you confident you will have access to quality medical care?

1 Yes

2 No

**Q11**.

**In the next month**, are you confident that if you had a high medical bill you or your health insurance plan (if any) would be able to pay for it? Think about a medical bill due to a major surgery, for example.

1 Yes

2 No

**Q12.**

Is there a particular doctor’s office, clinic, health center, or other place that you **usually** go if you are sick or need advice about your health?

1.Yes

2. There is NO place

3. There is MORE THAN ONE place

[If Q12 = 2 then skip to Q14]

**Q13.**

What kind of place do you go to **most often** - a clinic, a doctor's office, an emergency room, or some other place?

1. Clinic or health center

2. Doctor's office or HMO

3. Hospital emergency room

4. Hospital outpatient department

5. Some other place

6. No one place most often

**Q14.**

**In the last 12 months**, were you delayed in getting medical care, tests, or treatments that you or a doctor believed necessary?

1 Yes

2 No

[If yes]

Which of these best describes the main reason you were delayed in getting medical care, tests, or treatments that you or a doctor believed necessary?

1. Couldn’t afford care
2. Insurance company wouldn’t approve, cover, or pay for care
3. Doctor refused to accept my insurance plan
4. Problems getting to doctor’s office
5. Couldn’t get time off work
6. Didn’t know where to go to get care
7. Was refused services
8. Didn’t have time or took too long
9. Other _________________________

**Q15**.

**In the last 12 months**, have you seen or talked to any of the following health care providers about your own health?

...A mental health professional such as a psychiatrist, psychologist, psychiatric nurse, or clinical social worker.

1 Yes

2 No

...A medical doctor or nurse practitioner in general practice, family medicine, or internal medicine who treats a variety of illnesses?

1 Yes

2 No

...A medical specialist (other than a psychiatrist) such as a cardiologist, gastroenterologist or orthopedist?

1 Yes

2 No

**Q16.**

Are you **currently** taking any medication prescribed by a doctor or other health professional?

1 Yes

2 No

[If YES, ask Q17]

**Q17**.

Are you **currently** taking any prescribed medication to manage anxiety, depression, or another emotional problem?

1 Yes

2 No

**Q18.**

Do you have health insurance coverage?

1 Yes

2 No

Which of the following best describes your current health insurance or health coverage plan?

Please check all that apply.

1. Insurance through my or my spouse’s/partner’s employer/union

2. Retiree Insurance through my or my spouse’s/partner’s former employer/union

3. Private insurance purchased directly from an insurance company or through a state or federal exchange marketplace

4. Medicare

5. Medicaid, Medical Assistance or any kind of government assistance plan for those with low incomes or a disability

6. Tricare or other military health care

7. VA, including CHAMPVA or VA Care

8. Other program:_____________

9. No coverage

**Q19.**

Has a doctor or other health professional **ever** told you that you had (check all that apply)

- 1. diabetes
  2. heart disease
  3. arthritis
  4. asthma
  5. cancer
  6. memory problems
  7. other ____________

**Q20.**

Which of the following word belongs to the same category as **red**?

1. Fast
2. Right
3. Orange
4. Important
5. Whole

**Q21. Word recall task**

Next, we’ll display a total of 10 words, one at a time, and ask you to recall as many as you can. We have purposely made the list long so that it will be difficult for anyone to recall all the words. Most people recall just a few. Please pay careful attention as the words are displayed because they will not be repeated. After all of the words have been displayed, we will ask you to type as many of the words as you can recall, in any order.

Please select "Next" when you are ready to begin

WORD LIST:

HOTEL

RIVER

TREE

SKIN

GOLD

MARKET

PAPER

CHILD

KING

BOOK

Now please type the words you can recall in the box below, separating each word with a space. When you no longer recall any new word, please hit next.

**INTRO 3: The next part of this survey will ask some general questions about you**

**Q22.**

What is your gender?

1. Female
2. Male

**Q23.**

What is your race? Check all that apply.

1. White
2. Black or African American
3. American Indian or Alaska Native
4. Asian
5. Other: SPECIFY _____________________

Are you Hispanic, Latino, or of Spanish origin?

1. Yes
2. No

**Q24.**

In which state are you currently residing?

1 Alaska (AK)

…

52 Puerto Rico

[drop down menu]

**Q25.**

Are you now married, widowed, divorced, separated or never married?

- 1. Married (Spouse lives with me)
  2. Married (Spouse lives elsewhere)
  3. Separated
  4. Divorced
  5. Widowed
  6. Never Married

**Q26.**

What is the highest level of school that you have completed or the highest degree you

have received?

1 Less than 1st grade

2 1st, 2nd, 3rd or 4th grade

3 5th or 6th grade

4 7th or 8th grade

5 9th grade

6 10th grade

7 11th grade

8 12th grade - no diploma

9 High school graduate - high school diploma or the equivalent (for example GED)

10 Some college but no degree

11 Associate degree in college - Occupational/vocational program

12 Associate degree in college - - Academic program

13 Bachelor’s degree (For example: BA, AB, BS)

14 Master’s degree (For example: MA, MS, MEng, MEd, MSW, MBA)

15 Professional School Degree (For example: MD,DDS,DVM,LLB,JD)

16 Doctorate degree (For example: PhD, EdD)

**Q27.**

What is your labor force status? Please choose all that apply.

1 Currently working

2 On sick or other leave

3 Unemployed - on layoff

4 Unemployed – looking for work

5 Retired

6 Disabled

7 Other

**Q28.**

How many children do you have (please include all children deceased or alive)

- 1. 1
  2. 2
  3. 3
  4. 4
  5. 5 or more
  6. I never had any children

**Q29. Delayed Word recall task**

A little while ago, we displayed a list of words and you typed the ones you could remember. Please type any of the words that you still remember now, separating each word with a space.

**Q30.**

Which category represents the total combined income of all members of your family (living

in your house) during the past 12 months? This includes money from jobs, net income

from business, farm or rent, pensions, dividends, interest, Social Security payments and

any other monetary income received by members of your family who are 15 years of age

or older.

1 Less than $5,000

2 5,000 to 7,499

3 7,500 to 9,999

4 10,000 to 12,499

5 12,500 to 14,999

6 15,000 to 19,999

7 20,000 to 24,999

8 25,000 to 29,999

9 30,000 to 34,999

10 35,000 to 39,999

11 40,000 to 49,999

12 50,000 to 59,999

13 60,000 to 74,999

14 75,000 to 99,999

15 100,000 to 149,999

16 150,000 or more

[if younger than 65 ask Q31]

**Q31.**

When you turn 65 and are eligible for Medicare do you expect …

Better access to medical care (doctors, hospitals, prescription drugs)

Yes

No

Paying less out-of-pocket for medical care

Yes

No

Protection against very high medical bills

Yes

No

[if older than 65 ask Q32]

**Q32.**

If you have Medicare, has it met your expectations in terms of …

Better access to medical care (doctors, hospitals, prescription drugs)

Yes

No

Don’t have Medicare [then skip next two questions]

Paying less out-of-pocket for medical care

Yes

No

Protection against very high medical bills

Yes

No

[If marked Medicare in question 19, then go to Q33. If not, then skip to Q36]

**Q33.**

People who qualify for Medicare can obtain their medical coverage for things like doctor or hospital care in different ways. Which of the following describes your Medicare coverage?

a. Traditional Medicare

b. Private Medicare Advantage Plan

c. Don't know

[If Q33= private medicare advantage plan then skip to Q35]

**Q34.**

Medicare supplemental or Medigap policies are designed to cover the costs of doctor visits or hospital care that are not covered by Medicare. Are you covered by a supplemental Medigap policy you bought on your own or through a previous or current employer (of yours or your spouses)?

Yes

No

Don’t know

**Q35.**

Do you have prescription drug coverage through Medicare Part D? People get this type of coverage either through a Medicare health plan, such as a Medicare HMO, that covers prescription drugs, or through a separate Medicare prescription drug plan.

Yes

No

Don’t know

**Q36.**

Have you ever received in the mail a “Welcome to Medicare” letter? The “Welcome to Medicare” letter explains Medicare benefits, coverage choices, and how to enroll on Medicare.

1. Yes

2. No

**Q37.**

Have you ever received any other information, either through the mail or electronically, about Medicare benefits?

1. Yes

2. No

[If yes]

**Q38.**

Was the information from:

1. The Center for Medicare and Medicaid Services (CMS)

2. Your or your spouse's employer

3. A Medicare Advantage plan or private health insurance company

4. other ___________________

INTRO: The next questions ask about your life **when you were 55 years old.**

**Q39.**

**When you were 55,** were you married, widowed, divorced, separated or never married?

1. Married (Spouse lives with me)

2. Married (Spouse lives elsewhere)

3. Separated

4. Divorced

5. Widowed

6. Never Married

**Q40.**

**When you were 55,** what was your labor force status? Please choose all that apply.

1 Working

2 On sick or other leave

3 Unemployed - on layoff

4 Unemployed – looking for work

5 Retired

6 Disabled

7 Other

**Q41.**

**When you were 55,** how many of your children lived at home with you?

- - 1. 0
    2. 1
    3. 2
    4. 3
    5. 4
    6. 5 or more
    7. I never had any children

**Q42**.

**When you were 55,** did you have health insurance coverage?

1 Yes

2 No

**When you were 55,** which of the following best described your health insurance or health coverage plan?

Please check all that apply.

1. Insurance through my or my spouse’s/partner’s employer/union

2. Retiree Insurance through my or my spouse’s/partner’s former employer/union

3. Private insurance purchased directly from an insurance company or through a state or federal exchange marketplace

4. Medicare

5. Medicaid, Medical Assistance or any kind of government assistance plan for those with low incomes or a disability

6. Tricare or other military health care

7. VA, including CHAMPVA or VA Care

8. Other program:_____________

9. No coverage

**Q43.**

**By age 55,** had you been told by a doctor or other health professional that you had hypertension, also called high blood pressure?

1 Yes

2 No

**Q44.**

**By age 55**, had you been told by a doctor or other health professional that you had:

Coronary heart disease?

1 Yes

2 No

Diabetes or sugar diabetes? Do not include diabetes during pregnancy.

1 Yes

2 No

Cancer or a malignancy of any kind?

1 Yes

2 No

Some form of arthritis, rheumatoid arthritis, gout, lupus, or fibromyalgia?

1 Yes

2 No

Depression?

1 Yes

2 No

**Q45. Figure Identification Task**

**INTRO: The last few questions ask you about who lives with you and your income according to your latest tax forms.**

**Q46.**

Do you live with a spouse?

- - - 1. Yes
      2. No

[If no to Q46 then go to Q48]

**Q47.**

Do you and your spouse file your taxes jointly?

- - - - 1. Yes
        2. No

**Q48.**

Did you receive any supplemental security income last year?

Yes

No

**Q49.**

Did you receive any social security income last year?

1. Yes

2. No

**Q50.**

Did you receive any disability benefits last year?

1. Yes

2. No

**Q51.**

What is your best estimate of the amount you reported as Adjusted Gross Income (AGI) on your US 1040 tax form last year? Your Adjusted Gross Income is approximately your earnings from wages, tips plus any taxable interest or dividends, taxable refunds or credits, alimony received, taxable pension or annuities, unemployment compensation, taxable social security benefits. Please estimate your AGI to the nearest $1,000.

Less than $6,000

$6,000-6,999

$7,000-7,999

$8,000-8,999

$9,000-9,999

$10,000-10,999

$11,000-11,999

$12,000-12,999

$13,000-13,999

$14,000-14,999

$15,000-15,999

$16,000-16,999

$17,000-17,999

$18,000-18,999

$19,000-19,999

$20,000-20,999

$21,000-21,999

$22,000-22,999

$23,000-23,999

$24,000-24,999

$25,000-25,999

$26,000-26,999

$27,000-27,999

$28,000-28,999

$29,000-29,999

$30,000-30,999

$31,000-31,999

$32,000-32,999

$33,000-33,999

$34,000-34,999

$35,000-35,999

$36,000-36,999

$37,000-37,999

$38,000-38,999

$39,000-40,000

More than $40,000

Don’t know

**Q52.**

How many children (ages 18 and below) did you claim as dependents on your Form 1040 last year?

Drop menu 0 to 9 and more than 10

Don’t know.

[If don’t know to Q52 ask]

How many children (ages 18 and below) currently live with you?

Drop menu 0 to 9 and more than 10

**[Final Screen showing scores in word recall and figure ID]**
