# Supplementary material for: Varied and unexpected changes in the well-being of seniors in the United States amid the COVID-19 pandemic
Source: PLoS One. 2021 Jun 17;16(6):e0252962. doi: 10.1371/journal.pone.0252962 (PMC8211190; doi:10.1371/journal.pone.0252962)
Supplement: S2 File — (DOCX) [file pone.0252962.s002.docx]

**S2 File. Survey Instrument: Wave 2**

Notes to programmer are given in brackets [.] Questions in blue were asked in our baseline survey and should not be changed.

SCREEN 1:

INTRO: Welcome to this survey!

This survey asks you about your health and how you feel about your life.

Next, we will show you an information sheet about our study. Once you click next, you will be taken to the survey.

Thank you for your participation! We greatly value your input and hope that you will find this survey interesting.

**[SCREEN 2: DIFFERENT SCREEN** SHOW INFO SHEET IN SCROLL BOX**]**

SCREEN 3:

You might recognize some of the questions below from a previous survey you answered.

Some of the questions in this survey ask you about your past, about what happened and how you felt. Some ask about the recent past like yesterday and others about a more distant past like the past 2 weeks.

To help you, the time period for each question is shown in bold.

If you are taking this survey on a smartphone we recommend holding the screen horizontally (landscape mode)

| 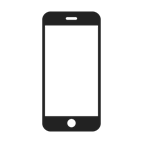 | 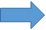 | 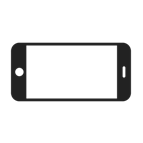 |
| --- | --- | --- |

**Q0.**

What is your date of birth? [drop down menu for each]

DD MM YYYY

[PLEASE LET RESPONDENTS CONTINUE NO MATTER WHAT THE AGE IS]

**Q1.**


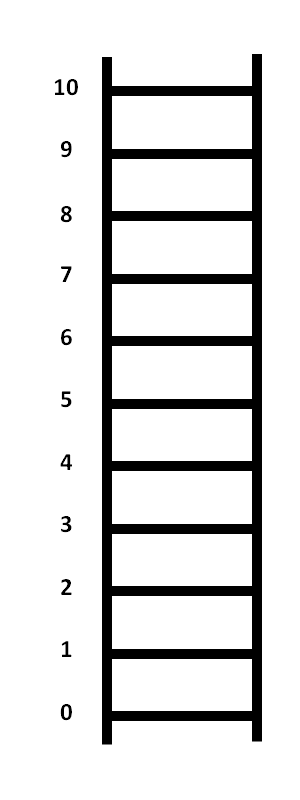
Please imagine a ladder with steps numbered from zero at the bottom to ten at the top. The top of the ladder represents the best possible life for you and the bottom of the ladder represents the worst possible life for you. On which step of the ladder would you say you personally feel you stand at this time?

10 Best possible

09

08

07

06

05

04

03

02

01

00 Worst possible

**Q2.**

In general, how satisfied are you with your life?

1. Very satisfied
2. Somewhat satisfied
3. Neither satisfied nor dissatisfied
4. Somewhat dissatisfied
5. Very dissatisfied

**Q3.**

[Please show one question per screen, keep the same order in which the questions were presented in the baseline]

Did you experience the following feelings during A LOT OF THE DAY **yesterday**? Yesterday, did you experience _____?

Q3.1. Enjoyment

Q3.2. Physical Pain

Q3.3. Worry

Q3.4.Sadness

Q3.5. Stress

Q3.6. Anger

Q3.7. Happiness

Response scale:

1Yes

2 No

**Q4.**

Did you worry about money **yesterday**?

1 Yes

2 No

**Q5.**

In general, would you say your health is:

1. Excellent
2. Very Good
3. Good
4. Fair
5. Poor

**Q6.**

In general, would you say your ***mental health*** is:

1. Excellent
2. Very Good
3. Good
4. Fair
5. Poor

**Q7.**

The following two questions ask about how you have been feeling in the **past 2 weeks**.

**Over the last 2 weeks**, how often have you been bothered by any of the following problems?

Little interest or pleasure in doing things

1. Nearly every day
2. More than half the days
3. Several days
4. Not at all

Feeling down, depressed, or hopeless

1. Nearly every day
2. More than half the days
3. Several days
4. Not at all

**INTRO 2: Now we will ask you some questions about your medical care use**

**Q8.**

**In the next month,** are you confident you will have access to quality medical care?

1 Yes

2 No

**Q9**.

**In the next month**, are you confident that if you had a high medical bill you or your health insurance plan (if any) would be able to pay for it? Think about a medical bill due to a major surgery, for example.

1 Yes

2 No

**Q9a.** **During the past 12 months**, have you had any problems paying bills?

1. Yes
2. No

[If yes, ask Q9b and c

**Q9b.** **During the past 12 months**, have you had any problems paying ***medical*** bills?

1. Yes
2. No

**Q9c.** Because of problems paying bills **during the past 12 month**, have you been contacted by a collection agency?

1. Yes
2. No

**Q10.**

Are you **currently** taking any medication prescribed by a doctor or other health professional?

1 Yes

2 No

[If YES, ask Q11]

**Q11**.

Are you **currently** taking any prescribed medication to manage anxiety, depression, or another emotional problem?

1 Yes

2 No

**Q12.**

Do you have health insurance coverage?

1 Yes

2 No

Which of the following best describes your current health insurance or health coverage plan?

Please check all that apply.

1. Insurance through my or my spouse’s/partner’s employer/union

2. Retiree Insurance through my or my spouse’s/partner’s former employer/union

3. Private insurance purchased directly from an insurance company or through a state or federal exchange marketplace

4. Medicare

5. Medicaid, Medical Assistance or any kind of government assistance plan for those with low incomes or a disability

6. Tricare or other military health care

7. VA, including CHAMPVA or VA Care

8. Other program:_____________

9. No coverage

**Q13.**

[Randomize the order in which answers 1-7 are presented]

Has a doctor or other health professional **ever** told you that you had (check all that apply)

- 1. diabetes
  2. heart disease
  3. arthritis
  4. respiratory or lung disease such as asthma or COPD
  5. cancer
  6. memory problems
  7. depression or anxiety
  8. other ____________
  9. Never been told I have any of these conditions

**Q14. Word recall task**

Next, we’ll display a total of 10 words, one at a time, and ask you to recall as many as you can. We have purposely made the list long so that it will be difficult for anyone to recall all the words. Most people recall just a few. Please pay careful attention as the words are displayed because they will not be repeated. After all of the words have been displayed, we will ask you to type as many of the words as you can recall, in any order.

Please select "Next" when you are ready to begin

WORD LIST:

SKY

OCEAN

FLAG

DOLLAR

WIFE

MACHINE

HOME

EARTH

COLLEGE

BUTTER

Now please type the words you can recall in the box below, separating each word with a space. When you no longer recall any new word, please hit next.

**INTRO 3: The next part of this survey will ask some general questions about you**

**Q15.**

In which state are you currently residing?

1 Alaska (AK)

…

52 Puerto Rico

[drop down menu]

**Q15a.** What is the ZIP code of your current residence?

[text box with the relevant number of boxes (5?) – please add error message if they don’t fill all the boxes]

**Q15b**.Have you moved since **November 2019**?

1 Yes

2 No

If Yes ask q15c

**Q15c**. What is the ZIP code of the residence were you were living in **November 2019**?

[text box with the relevant number of boxes (5?) – please add error message if they don’t fill all the boxes]

**Q16.**

Which of the following best describes the place where you live now…

1. a large city

2. a suburb near a large city

3. a small city or town

4. a rural area

**Q17.**

What is your labor force status? Please choose all that apply.

1 Currently working

2 On sick or other leave

3 Unemployed - on layoff

4 Unemployed – looking for work

5 Retired

6 Disabled

7 Other

If Q17 == 1 or 2, ask Q17a-f

**Q17a.**

Do you have the kind of job where working from home is an option, if required?

- - 1. Yes
    2. No
    3. Unsure

**Q17b.** Have you changed employers since **November 2019**?

1 Yes

2 No

[If no, ask questions Q17c and d. If Yes, ask questions Q17 e and f]

**Q17c.**

Are you employed by government, by a private company, a nonprofit organization, or self-employed or working in a family business?

1 Government

2 Private for profit company

3 Non profit organization including tax exempt and charitable organizations

4 Self employed

5 Working in family business

**Q17d.**

Counting all locations where your employer (or you if self-employed) operate, what is the total number of persons who work for your employer/you?

1 I am self-employed and I do not employ anyone

2 1-10

3 10-49

4 50-99

5 100-499

6 500-999

7 1,000+

**Q17e.**

Think about your employer in November 2019. Were you employed by government, by a private company, a nonprofit organization, or self-employed or working in a family business?

1 Government

2 Private for profit company

3 Non profit organization including tax exempt and charitable organizations

4 Self employed

5 Working in family business

**Q17f.**

Think about your employer in November 2019. Counting all locations where your employer (or you if self-employed) operated, what was the total number of persons who worked for your employer/you?

1 I was self-employed and I did not employ anyone

2 1-10

3 10-49

4 50-99

5 100-499

6 500-999

7 1,000+

**Q18.**

How many people live at home with you?

- 1. I live alone
  2. 1
  3. 2
  4. 3
  5. 4
  6. 5
  7. 6 or more

**Q18a.**

**In November 2019**, how many people lived at home with you?

- 1. I lived alone
  2. 1
  3. 2
  4. 3
  5. 4
  6. 5
  7. 6 or more

**Q19.**

How do you see yourself: are you a person who is generally willing to take risks or do you try to avoid taking risks?

[The question should be followed by a clickable horizontal boxes with the numbers 0 through 10 in them. To the left of 0 should be the text “completely unwilling to take risks”, and to the right of 10 should be the text, “very willing to take risks”.]

**Q20.**

How willing are you to give up something that is beneficial for you today in order to benefit more from that in the future?

[The question should be followed by a clickable horizontal boxes with the numbers 0 through 10 in them. To the left of 0 should be the text “completely unwilling to do so”, and to the right of 10 should be the text, “very willing to do so”.]

**Q21.**

How well does the following statement describe you as a person?

“I tend to postpone things even though it would be better to get them done right away.”

[The question should be followed by a clickable horizontal boxes with the numbers 0 through 10 in them. To the left of 0 should be the text “does not describe me at all”, and to the right of 10 should be the text, “describes me perfectly”.]

**Q22.**

You will find below a series of statements which describe how people may react to the uncertainties of life. Please use the scale below to describe to what extent each item is characteristic of you. Please circle a number (1 to 5) that describes you best.

|  | Not at all characteristic of me | A little characteristic of me | Somewhat characteristic of me | Very characteristic of me | Entirely characteristic of me |
| --- | --- | --- | --- | --- | --- |
| 1. Unforeseen events upset me greatly. | 1 | 2 | 3 | 4 | 5 |
| 2. It frustrates me not having all the information I need. | 1 | 2 | 3 | 4 | 5 |
| 3. Uncertainty keeps me from living a full life. | 1 | 2 | 3 | 4 | 5 |
| 4. One should always look ahead so as to avoid surprises. | 1 | 2 | 3 | 4 | 5 |
| 5. A small unforeseen event can spoil everything, even with the best of planning. | 1 | 2 | 3 | 4 | 5 |
| 6. When it’s time to act, uncertainty paralyses me. | 1 | 2 | 3 | 4 | 5 |
| 7. When I am uncertain I can’t function very well. | 1 | 2 | 3 | 4 | 5 |
| 8. I always want to know what the future has in store for me. | 1 | 2 | 3 | 4 | 5 |
| 9. I can’t stand being taken by surprise. | 1 | 2 | 3 | 4 | 5 |
| 10. The smallest doubt can stop me from acting. | 1 | 2 | 3 | 4 | 5 |
| 11. I should be able to organize everything in advance. | 1 | 2 | 3 | 4 | 5 |
| 12. I must get away from all uncertain situations. | 1 | 2 | 3 | 4 | 5 |

**Q23.** Big 5 personality

Instead of asking them to write a number, let’s say:

Here are a number of personality traits that may or may not apply to you. Please indicate the extent to which you agree or disagree with each statement. You should rate the extent to which the pair of traits applies to you, even if one characteristic applies more strongly than the other.

**Q24.**

Below we would like to ask your opinion about how likely an event might be. When we ask a question we'd like for you to give us a number from 0 to 100, where "0" means that you think there is absolutely no chance, and "100" means that you think the event is absolutely sure to happen. For example, no one can ever be sure about tomorrow's weather, but if you think that rain is very unlikely tomorrow, you might say that there is a 10 percent chance of rain. If you think there is a very good chance that it will rain tomorrow, you might say that there is an 80 percent chance of rain

On a scale of 0 to 100 percent, what is the chance that you will be doing any work for pay **by this time next year**? If you’re not sure, please give your best guess.

[Include ruler]

RANGE 0 100

Please enter a number between 0% and 100%

**Q25.** Do you have any money in a retirement or tax deferred savings account through either a current or past employer or union?

- - 1. Yes
    2. No
    3. Unsure

[If yes]

**Q25a.**

Some retirement plans base benefits on a formula involving age, years of service and salary, often called a defined benefit plan. Some plans base benefits on how much money has accumulated in a person's retirement account, often called a defined contribution plan. Other plans use both ways of setting benefits.

Think about the plan where you have the largest amount of money saved for retirement. What type of plan is it?

1. DEFINED BENEFIT(FORMULA)

2. DEFINED CONTRIBUTION (ACCOUNT)

3. BOTH TYPES

Unsure

**Q26.**

**In the last month**, did you provide care for or look after an adult or child who cannot care for themselves?

YES

NO

**Q26a.**

Who was it that you provided care for or looked after? USE RELATIONSHIP CODES FOR Q1. [drop down]

**Q26b.**

**In November 2019**, were you already providing care for this person?

1 Yes

2 No

**Q27.**

Do any of your close family members or friends live in a nursing home or assisted living facility?

- - - 1. Yes
      2. No

**Q28. Delayed Word recall task**

A little while ago, we displayed a list of words and you typed the ones you could remember. Please type any of the words that you still remember now, separating each word with a space.

**Q29.**

Which of the following words belongs to the same category as **bicycle**?

1. Little
2. Window
3. Motorcycle
4. Rain
5. Tender

**Q30. Figure Identification Task**

**Q31. [4 questions on the same screen]**

Please respond to each question or statement by marking one box per row.

**In the past 7 days…**

My sleep quality was ….

- 1. Very poor
  2. Poor
  3. Fair
  4. Good
  5. Very good

**In the past 7 days…**

My sleep was refreshing…

I had a problem with my sleep…

I had difficulty falling asleep…

- 1. Not at all
  2. A little bit
  3. Somewhat
  4. Quite a bit
  5. Very much

**Q32. [3 questions on the same screen]**

Think about how you have been feeling in the **past 7 days.**

1. In the **past 7 days**, how often did you feel that you lack companionship?

2. In the **past 7 days**, how often did you feel left out?

3. In the **past 7 days**, how often did you feel isolated from others?

Answers for each are:

1. Hardly Ever

2. Some of the Time

3. Often

**Q33.**

The following two questions ask about how you have been feeling in the **past 2 weeks**.

**Over the last 2 weeks**, how often have you been bothered by any of the following problems?

Feeling nervous, anxious or on edge

1. Nearly every day
2. More than half the days
3. Several days
4. Not at all

Not being able to stop or control worrying

1. Nearly every day
2. More than half the days
3. Several days
4. Not at all

**INTRO: In the last questions, we will ask you what you are thinking and feeling about the coronavirus (COVID-19)**

**Q34.**

Have you heard of the coronavirus (COVID-19)?

- - 1. Yes
    2. No
    3. Unsure

**[if No or Unsure then show]**

The coronavirus or COVID-19 is a new disease with flu-like symptoms that is spreading across the world.

**Q35.**

Do you think the coronavirus is a real threat or blown out of proportion?

- - 1. A real threat
    2. Blown out of proportion
    3. Unsure

**Q36.**

Have you been tested for the coronavirus (COVID-19)?

- - 1. Yes, I tested positive
    2. Yes, I tested negative
    3. No, I have not been tested

**[If Q36 “yes, I tested positive” skip Q37]**

**Q37a.**

Do you think you’ve been infected with the coronavirus (COVID-19)?

- - 1. Yes
    2. No
    3. Unsure

**[If Q37 “No” skip Q37b]**

**Q37b.**

Did you try to get tested for the coronavirus (COVID-19)?

- - 1. Yes but I was unable to get tested
    2. Yes but I’m still waiting for the results
    3. No
    4. Unsure

**Q38a.**

Do you personally know anyone who has been diagnosed with the coronavirus (COVID-19)?

- - 1. Yes
    2. No
    3. Unsure

If Q38a = YES, then ask Q38b.

**Q38b.**

Do any of your close relatives or friends have been diagnosed with the coronavirus (COVID-19)?

- - 1. Yes
    2. No
    3. Unsure

**Q39.** How many people in your state of residence are currently infected with the coronavirus (COVID-19)? If you’re not sure, please give your best guess. Please enter numbers only.

**_________________ (open ended)**

**Q40. In the last month**, were you delayed in getting medical care, tests, or treatments that you or a doctor believed necessary?

- - - 1. Yes
      2. No

[If yes]

**Q40a.**

[Order of answers is randomized, other always at the bottom]

Which of these best describes the main reason you were delayed in getting medical care, tests, or treatments that you or a doctor believed necessary?

 1.      Couldn’t afford care

2.      Insurance company wouldn’t approve, cover, or pay for care

3.      Doctor refused to accept my insurance plan

4.      Problems getting to doctor’s office

5.      Worried that would get infected with COVID-19 at doctor’s office

6.      Couldn’t get time off work

7.      Didn’t know where to go to get care

8.      Was refused services

9.      Didn’t have time or took too long

10. Doctor’s office was closed

11.  Other _________________________

**Q41a.** If you needed help, how many people do you feel you could call who would help you?

- 1. 1
  2. 2
  3. 5
  4. 4
  5. 5 or more
  6. I have no one I could call.

**Q41b.**

Have you lost your job or a significant part of your income because of the coronavirus (COVID-19)?

1. Yes, I lost my job
2. Yes, I did not lose my job but I lost a significant part of my income
3. No

Next we would like to ask your opinion about how likely you think various events might be. When we ask a question we'd like for you to give us a number from 0 to 100, where "0" means that you think there is absolutely no chance, and "100" means that you think the event is absolutely sure to happen. For example, no one can ever be sure about tomorrow's weather, but if you think that rain is very unlikely tomorrow, you might say that there is a 10 percent chance of rain. If you think there is a very good chance that it will rain tomorrow, you might say that there is an 80 percent chance of rain.

**Q42.**

On a scale of 0 to 100 percent, what is the chance that you will get the coronavirus (COVID-19) in the **next three months**? If you’re not sure, please give your best guess.

RANGE 0 100

Please enter a number between 0% and 100%

**Q43.**

If you do get the coronavirus (COVID-19), what is the percent chance you will die from it? If you’re not sure, please give your best guess.

RANGE 0 100

Please enter a number between 0% and 100%

**Q44.**

On a scale of 0 to 100 percent, what is the chance that someone you know will get the coronavirus (COVID-19) in the **next three months**? If you’re not sure, please give us your best guess.

RANGE 0 100

Please enter a number between 0% and 100%

**Q45.**

If someone the same age as you gets the coronavirus (COVID-19), what is the percent chance they will die from it? If you’re not sure, please give us your best guess.

RANGE 0 100

Please enter a number between 0% and 100%

[Ask if Q46 currently working Q17= 1 or 2 (currently working or on leave) ]

**Q46.**

The coronavirus (COVID-19) may cause economic challenges for some people regardless of whether they are actually infected.

What is the percent chance that you will lose your job because of the coronavirus (COVID-19) within the **next three months**?

RANGE 0 100

Please enter a number between 0% and 100%

**Q47.**

The coronavirus (COVID-19) may cause economic challenges for some people regardless of whether they are actually infected.

What is the percent chance you will run out of money because of the coronavirus (COVID-19) in the **next three months**?

RANGE 0 100

Please enter a number between 0% and 100%

**Q48.**

[Order of answers is randomized, I’ve done nothing different always in the bottom]

Which of the following have you done in the **past 7 days** to keep yourself safe from the coronavirus (COVID-19) in addition to what you normally do?

**Only consider actions that you took or decisions that you made personally. Mark all that apply.**

1. Worn a face mask
2. Washed hands with soap or used hand sanitizer several times per day
3. Canceled or postponed air travel
4. Canceled or postponed work or school activities
5. Canceled or postponed personal or social activities
6. Visited a doctor
7. Canceled a doctor’s appointment
8. Stockpiled food or water
9. Avoided contact with people who could be high-risk
10. Avoided public spaces, gatherings, or crowds
11. Avoided eating at restaurants
12. Worked or studied at home
13. Self-isolated at home
14. Avoided interacting with own children or/and grandchildren
15. I’ve done nothing differently

[If they mark self-isolated at home, ask Q49a]

**Q48a.** How long have you been self-isolating at home because of the coronavirus (COVID-19)?

_____ [open ended, allow numbers only] days/weeks [drop down menu]

**In the past 7 days**, did the coronavirus (COVID-19) keep you from

|  | Yes | No |
| --- | --- | --- |
| Q49. visiting in person with friends and family not living with you |  |  |
| Q51. attending religious services |  |  |
| Q53. participating in clubs, classes, or other organized activities |  |  |
| Q55. exercising (i.e. walking, working out, running, biking, swimming, playing a sport, etc.)? |  |  |
| Q57. getting your usual help with household chores or other daily activities (e.g. cleaning, cooking, laundry, child or elderly care)? |  |  |

Would you say the following activities are very important, somewhat important, or not so important to you?

|  | Very Important | Somewhat Important | Not Important |
| --- | --- | --- | --- |
| Q50. visiting in person with friends and family not living with you |  |  |  |
| Q52. attending religious services |  |  |  |
| Q54. participating in clubs, classes, or other organized activities |  |  |  |
| Q56. exercising (i.e. walking, working out, running, biking, swimming, playing a sport, etc.)? |  |  |  |
| Q58. getting your usual help with household chores or other daily activities (e.g. cleaning, cooking, laundry, child or elderly care)? |  |  |  |

**Q59.**

[Order of answers 1-7 is randomized]

Have you changed your plans with respect to any of the below because of the coronavirus (COVID-19)? Please mark all that apply.

- - 1. Travel
    2. Work
    3. Retirement
    4. Health treatments and procedures
    5. Residential move
    6. Real estate or vehicle purchase or sale
    7. Big ticket purchase or sale
    8. Other ______________________
    9. I have not changed any of my plans

**Q60.**

Please tell us what your primary source of information about the coronavirus (COVID-19) is. **Please be specific about the sources. For example, if your primary source of information is TV, please tell us which channel; if it is a newspaper, please tell us which newspaper; if internet or social media, please tell us which site.**

________________________ [open ended]

**Q60b.**

Regarding the answer to the prior question, how many minutes or hours a day do you spend with that media outlet?

____ Hours ____Minutes

**[Final Screen showing scores in word recall and figure ID]**

Thank you for participating in our survey!

We will be monitoring people’s feelings and opinions in the coming months and we might contact you again for another interview. Your answers are very important to us. We appreciate your cooperation!
