# Supplementary material for: Varied and unexpected changes in the well-being of seniors in the United States amid the COVID-19 pandemic
Source: PLoS One. 2021 Jun 17;16(6):e0252962. doi: 10.1371/journal.pone.0252962 (PMC8211190; doi:10.1371/journal.pone.0252962)
Supplement: S6 File — (DOCX) [file pone.0252962.s006.docx]

This study, which is funded by the U.S. National Institutes of Health, is being conducted on behalf of Dr. Silvia Barcellos at the University of Southern California. More details below.

**Purpose of the Study**

To document how the health and well-being of Americans is changing over time. We will ask you questions about your physical and mental health.

**What you will do in this study**

If you agree to participate, you will play games and answer an online survey. That takes approximately 25 minutes.

**Risks and Benefits**

There are no risks associated with this study. You will not directly benefit from your participation, but the researchers hope to understand the factors affecting the health and well-being of Americans.

**Compensation**

You will receive your usual survey reward if you complete the survey. You will also receive a $10 extra reward if you complete the survey.

**Confidentiality**

You will not be individually identified and your responses will be used for analyses only.

**Participation and Withdrawal**

As with all Dynata surveys, your response to this survey, or any individual question on the survey, is completely voluntary.

**Investigator’s Contact Information**

If you have any questions or concerns about the research, contact Silvia Barcellos, University of Southern California, 635 Downey Way, VPD Room 505F, Los Angeles, CA 90089, USA. Email: [silvia.barcellos@usc.edu](mailto:silvia.barcellos@usc.edu). Phone: 1-213- 821-2732.

**IRB Contact Information**

If you have any questions about your rights as a research participant, please contact the University of Southern California Institutional Review Board at (323) 442-0114 or email irb@usc.edu.
