# Supplementary material for: Varied and unexpected changes in the well-being of seniors in the United States amid the COVID-19 pandemic
Source: PLoS One. 2021 Jun 17;16(6):e0252962. doi: 10.1371/journal.pone.0252962 (PMC8211190; doi:10.1371/journal.pone.0252962)
Supplement: S1 Table — (PDF) [file pone.0252962.s007.pdf]

S1 Table. Predictors of only Wave 1 Participation

	Wave 1 only completition: 0.258		
age	0.152	-0.026	-0.025
	(0.059)	(0.039)	(0.039)
age squared	-0.001	0.0002	0.0002
	(0.0005)	(0.0003)	(0.0003)
female	0.052	0.020	0.019
	(0.006)	(0.004)	(0.004)
minority	0.051	0.033	0.035
	(0.009)	(0.006)	(0.006)
insured at age 55	-0.052	0.013	0.014
	(0.011)	(0.008)	(0.008)
hypertension at age 55	0.013	0.002	0.002
	(0.006)	(0.004)	(0.004)
heart disease at age 55	0.056	0.023	0.022
	(0.014)	(0.009)	(0.009)
diabetes at age 55	0.033	0.001	0.001
	(0.009)	(0.006)	(0.006)
cancer at age 55	-0.006	-0.001	-0.001
	(0.011)	(0.007)	(0.007)
arthritis at age 55	0.034	0.005	0.004
	(0.006)	(0.004)	(0.004)
depression age age 55	0.102	0.043	0.036
	(0.008)	(0.005)	(0.005)
married at age 55	-0.049	-0.003	-0.004
	(0.008)	(0.007)	(0.007)
divorced at age 55	-0.007	0.007	0.007
	(0.010)	(0.006)	(0.006)
working at age 55	0.007	0.009	0.009

S1 Table. Predictors of only Wave 1 Participation

	Wave 1 only completition: 0.258		
	(0.007)	(0.005)	(0.005)
children at age 55	-0.007	0.001	0.001
	(0.001)	(0.001)	(0.001)
above 90th COVID death rate		-0.002	-0.003
		(0.008)	(0.008)
death rate missing		0.679	0.678
		(0.008)	(0.008)
less than college		0.006	0.006
		(0.004)	(0.004)
married		-0.016	-0.015
		(0.006)	(0.006)
non_expansion		0.006	0.006
		(0.004)	(0.004)
children		-0.002	-0.002
		(0.002)	(0.002)
inc2 [30k, 50k)		-0.019	-0.017
		(0.006)	(0.006)
inc3 [50k, 75k)		-0.017	-0.015
		(0.006)	(0.006)
inc4 [75k, 100k)		-0.017	-0.014
		(0.007)	(0.007)
inc5 [100k, 150k)		-0.006	-0.004
		(0.007)	(0.007)
inc6 [150k+]		0.011	0.014
		(0.008)	(0.008)
working at wave 1		0.003	0.002
		(0.004)	(0.004)

S1 Table. Predictors of only Wave 1 Participation

	Wave 1 only completion: 0.258		
depressed at wave 1			0.019
			(0.007)
negative affect at wave 1			0.006
			(0.002)
positive affect at wave 1			0.005
			(0.003)
ladder at wave 1			-0.001
			(0.001)
self-rated health at wave 1			-0.001
			(0.002)
pain at wave 1			-0.001
			(0.004)
Constant	-4.236	0.934	0.889
	(1.900)	(1.252)	(1.252)
Observations	25,154	25,121	25,105
R-squared	0.03178	0.58117	0.58161

Estimates are from linear probability models of completion of only the wave 1 survey. Standard errors in parentheses
