# Supplementary material for: Varied and unexpected changes in the well-being of seniors in the United States amid the COVID-19 pandemic
Source: PLoS One. 2021 Jun 17;16(6):e0252962. doi: 10.1371/journal.pone.0252962 (PMC8211190; doi:10.1371/journal.pone.0252962)
Supplement: S2 Table — (PDF) [file pone.0252962.s008.pdf]

S2 Table. Changes in Well-Being across Waves by Demographic Characteristics

Panel A: Change in Proportion with Depressive Symptoms

	**All**	**Female**	**Male**	**No College**	**College**	**Retired**	**Not retired**	**Non-white**	**White**	**Married**	**Not Married**	**Income< 50,000**	**Income> = 50,000**
wave 2	0.014	0.024	0	0.019	0.012	0.017	0.011	0.019	0.014	0.016	0.011	0.013	0.015
	(0.008 - 0.021)	(0.015 - 0.032)	(-0.009 - 0.008)	(0.007 - 0.030)	(0.004 - 0.019)	(0.009 - 0.025)	(0.001 - 0.021)	(-0.003 - 0.041)	(0.007 - 0.020)	(0.009 - 0.023)	(-0.001 - 0.023)	(0.001 - 0.026)	(0.008 - 0.022)
Cons.	0.072	0.08	0.06	0.098	0.058	0.062	0.085	0.067	0.073	0.054	0.106	0.117	0.048
	(0.069 - 0.075)	(0.075 - 0.084)	(0.056 - 0.065)	(0.092 - 0.104)	(0.054 - 0.061)	(0.059 - 0.066)	(0.080 - 0.090)	(0.056 - 0.077)	(0.069 - 0.076)	(0.050 - 0.057)	(0.100 - 0.112)	(0.111 - 0.123)	(0.045 - 0.051)
Obs	33,815	20,678	13,137	12,183	21,632	19,402	14,413	3,015	30,800	21,838	11,977	11,822	21,993
R-sq.	0.712	0.711	0.713	0.728	0.694	0.706	0.717	0.68	0.715	0.692	0.728	0.727	0.686

Panel B: Change in Proportion Reporting Pain a Lot of the Day Yesterday

	**All**	**Female**	**Male**	**No College**	**College**	**Retired**	**Not retired**	**Non-white**	**White**	**Married**	**Not Married**	**Income< 50,000**	**Income> = 50,000**
wave 2	-0.051	-0.05	-0.052	-0.054	-0.049	-0.051	-0.05	-0.046	-0.051	-0.045	-0.061	-0.056	-0.048
	(-0.062 - -0.040)	(-0.064 - -0.036)	(-0.070 - -0.035)	(-0.072 - -0.035)	(-0.063 - -0.036)	(-0.065 - -0.037)	(-0.067 - -0.034)	(-0.080 - -0.012)	(-0.063 - -0.040)	(-0.059 - -0.032)	(-0.079 - -0.043)	(-0.074 - -0.038)	(-0.062 - -0.035)
Cons.	0.4	0.416	0.376	0.455	0.369	0.399	0.401	0.359	0.404	0.378	0.439	0.478	0.358
	(0.395 - 0.405)	(0.409 - 0.422)	(0.367 - 0.384)	(0.446 - 0.464)	(0.362 - 0.375)	(0.392 - 0.406)	(0.393 - 0.409)	(0.342 - 0.376)	(0.398 - 0.410)	(0.372 - 0.385)	(0.431 - 0.448)	(0.469 - 0.487)	(0.352 - 0.365)
Obs	33,817	20,680	13,137	12,184	21,633	19,405	14,412	3,014	30,803	21,839	11,978	11,823	21,994
R-sq.	0.739	0.745	0.729	0.743	0.734	0.734	0.746	0.756	0.738	0.731	0.752	0.759	0.722

S2 Table. Changes in Well-Being across Waves by Demographic Characteristics (continued)

Panel C: Change in Mean Negative Affect

	All	Female	Male	No College	College	Retired	Not retired	Non-white	White	Married	Not Married	Income< 50,000	Income> = 50,000
wave 2	0.225	0.274	0.148	0.19	0.245	0.241	0.204	0.144	0.233	0.253	0.175	0.154	0.263
	(0.197 - 0.254)	(0.237 - 0.312)	(0.106 - 0.191)	(0.142 - 0.238)	(0.210 - 0.280)	(0.204 - 0.278)	(0.160 - 0.248)	(0.047 - 0.241)	(0.204 - 0.263)	(0.218 - 0.288)	(0.127 - 0.223)	(0.105 - 0.203)	(0.229 - 0.298)
Cons.	1.088	1.193	0.924	1.185	1.034	0.996	1.213	0.959	1.101	1.005	1.241	1.295	0.977
	(1.074 - 1.102)	(1.174 - 1.211)	(0.903 - 0.946)	(1.162 - 1.209)	(1.016 - 1.051)	(0.977 - 1.014)	(1.192 - 1.235)	(0.911 - 1.007)	(1.086 - 1.116)	(0.987 - 1.022)	(1.217 - 1.265)	(1.271 - 1.319)	(0.960 - 0.994)
Obs	33,814	20,677	13,137	12,182	21,632	19,404	14,410	3,014	30,800	21,835	11,979	11,823	21,991
R-sq.	0.747	0.74	0.749	0.756	0.74	0.742	0.749	0.729	0.748	0.737	0.759	0.758	0.736

Panel D: Change in Mean Positive Affect

	All	Female	Male	No College	College	Retired	Not retired	Non-white	White	Married	Not Married	Income< 50,000	Income> = 50,000
wave 2	-0.104	-0.112	-0.092	-0.092	-0.111	-0.103	-0.105	-0.105	-0.104	-0.111	-0.092	-0.076	-0.119
	(-0.120 - -0.088)	(-0.133 - -0.090)	(-0.118 - -0.067)	(-0.121 - -0.064)	(-0.130 - -0.091)	(-0.124 - -0.082)	(-0.130 - -0.080)	(-0.162 - -0.048)	(-0.121 - -0.087)	(-0.130 - -0.092)	(-0.121 - -0.062)	(-0.106 - -0.046)	(-0.138 - -0.100)
Cons.	1.686	1.685	1.686	1.633	1.716	1.709	1.655	1.663	1.688	1.754	1.562	1.564	1.751
	(1.678 - 1.694)	(1.675 - 1.696)	(1.674 - 1.699)	(1.619 - 1.647)	(1.706 - 1.725)	(1.698 - 1.719)	(1.642 - 1.667)	(1.635 - 1.691)	(1.680 - 1.696)	(1.744 - 1.763)	(1.547 - 1.577)	(1.549 - 1.578)	(1.742 - 1.761)
Obs	33,812	20,675	13,137	12,182	21,630	19,402	14,410	3,013	30,799	21,834	11,978	11,821	21,991
R-sq.	0.72	0.711	0.733	0.727	0.714	0.71	0.73	0.711	0.72	0.702	0.732	0.729	0.706

S2 Table. Changes in Well-Being across Waves by Demographic Characteristics (continued)

Panel E: Change in Mean Cantril Ladder

	All	Female	Male	No College	College	Retired	Not retired	Non-white	White	Married	Not Married	Income< 50,000	Income> = 50,000
wave 2	-0.16	-0.188	-0.118	-0.121	-0.183	-0.187	-0.125	-0.088	-0.167	-0.209	-0.072	-0.028	-0.231
	(-0.193 - -0.128)	(-0.231 - -0.144)	(-0.167 - -0.068)	(-0.180 - -0.061)	(-0.221 - -0.144)	(-0.229 - -0.144)	(-0.176 - -0.074)	(-0.214 - 0.038)	(-0.201 - -0.134)	(-0.247 - -0.170)	(-0.132 - -0.012)	(-0.092 - 0.035)	(-0.268 - -0.194)
Cons.	7.297	7.247	7.376	7.021	7.453	7.47	7.065	7.25	7.302	7.614	6.719	6.567	7.689
	(7.281 - 7.313)	(7.226 - 7.268)	(7.352 - 7.400)	(6.992 - 7.050)	(7.434 - 7.472)	(7.449 - 7.490)	(7.040 - 7.090)	(7.188 - 7.312)	(7.285 - 7.318)	(7.595 - 7.633)	(6.690 - 6.749)	(6.536 - 6.599)	(7.671 - 7.707)
Obs	33,808	20,673	13,135	12,176	21,632	19,399	14,409	3,014	30,794	21,832	11,976	11,819	21,989
R-sq.	0.831	0.827	0.838	0.83	0.829	0.826	0.834	0.811	0.834	0.814	0.834	0.832	0.807

Panel F: Change in Mean Self-rated Health

	All	Female	Male	No College	College	Retired	Not retired	Non-white	White	Married	Not Married	Income< 50,000	Income> = 50,000
wave 2	0.004	0.003	0.005	0.006	0.003	0.015	-0.012	-0.004	0.004	0.005	0.002	-0.001	0.006
	(-0.008 - 0.016)	(-0.013 - 0.018)	(-0.013 - 0.024)	(-0.015 - 0.026)	(-0.012 - 0.017)	(-0.0003 - 0.031)	(-0.030 - 0.007)	(-0.045 - 0.037)	(-0.008 - 0.017)	(-0.010 - 0.019)	(-0.019 - 0.023)	(-0.023 - 0.020)	(-0.008 - 0.020)
Cons.	2.574	2.575	2.572	2.757	2.471	2.564	2.588	2.727	2.559	2.479	2.747	2.871	2.414
	(2.568 - 2.580)	(2.568 - 2.583)	(2.563 - 2.581)	(2.747 - 2.767)	(2.463 - 2.478)	(2.556 - 2.571)	(2.579 - 2.597)	(2.707 - 2.748)	(2.553 - 2.565)	(2.472 - 2.486)	(2.737 - 2.757)	(2.860 - 2.881)	(2.407 - 2.421)
Obs	33,819	20,681	13,138	12,184	21,635	19,406	14,413	3,015	30,804	21,840	11,979	11,824	21,995
R-sq.	0.912	0.908	0.917	0.906	0.911	0.909	0.915	0.899	0.913	0.908	0.913	0.908	0.905

Notes: The estimates here form the basis of Figure 1. For details, see notes to Figure 1 and equation 1 in the paper.
