# Supplementary material for: Varied and unexpected changes in the well-being of seniors in the United States amid the COVID-19 pandemic
Source: PLoS One. 2021 Jun 17;16(6):e0252962. doi: 10.1371/journal.pone.0252962 (PMC8211190; doi:10.1371/journal.pone.0252962)
Supplement: S3 Table — (PDF) [file pone.0252962.s009.pdf]

S3 Table. Predictors of the Z-score of changes in Well-Being across Waves

VARIABLES	depressive symptoms	pain	negative affect	positive affect	self-rated health	Cantril ladder
Extreme death rate	0.046	0.028	0.097	-0.045	0	-0.03
	(-0.012 - 0.103)	(-0.024 - 0.080)	(0.042 - 0.152)	(-0.097 - 0.007)	(-0.060 - 0.061)	(-0.093 - 0.034)
High chance of virus	0.033	0.014	0.132	-0.066	0.034	-0.089
	(0.002 - 0.064)	(-0.019 - 0.046)	(0.096 - 0.168)	(-0.099 - -0.034)	(0.000 - 0.069)	(-0.120 - -0.059)
High chance of dying	0.057	0	0.099	-0.047	0.063	-0.061
	(0.024 - 0.089)	(-0.031 - 0.032)	(0.065 - 0.133)	(-0.080 - -0.014)	(0.032 - 0.093)	(-0.092 - -0.029)
Kept from exercise	0.097	-0.031	0.079	-0.066	0.069	-0.057
	(0.062 - 0.131)	(-0.066 - 0.004)	(0.043 - 0.114)	(-0.101 - -0.032)	(0.037 - 0.101)	(-0.087 - -0.026)
Lost job/income	0.093	-0.049	0.11	-0.066	0.007	-0.199
	(0.044 - 0.143)	(-0.098 - -0.000)	(0.060 - 0.159)	(-0.111 - -0.021)	(-0.039 - 0.054)	(-0.252 - -0.146)
Female	0.074	0.007	0.1	-0.028	-0.01	-0.061
	(0.040 - 0.107)	(-0.023 - 0.038)	(0.067 - 0.133)	(-0.061 - 0.005)	(-0.041 - 0.021)	(-0.092 - -0.030)
No college	0.022	-0.011	-0.029	0.006	0.009	0.007
	(-0.014 - 0.058)	(-0.045 - 0.023)	(-0.063 - 0.005)	(-0.028 - 0.040)	(-0.024 - 0.042)	(-0.026 - 0.040)
Married	0.027	0.033	0.048	-0.014	-0.001	-0.056
	(-0.012 - 0.066)	(-0.000 - 0.066)	(0.011 - 0.085)	(-0.051 - 0.023)	(-0.037 - 0.034)	(-0.096 - -0.017)
Retired at wave1	0.034	-0.011	0.057	-0.018	0.052	-0.086
	(-0.000 - 0.068)	(-0.045 - 0.023)	(0.026 - 0.089)	(-0.050 - 0.013)	(0.013 - 0.090)	(-0.118 - -0.054)
Nonwhite	0.015	0.01	-0.048	-0.002	-0.014	0.031
	(-0.040 - 0.070)	(-0.038 - 0.058)	(-0.108 - 0.012)	(-0.062 - 0.057)	(-0.068 - 0.041)	(-0.032 - 0.094)

S3 Table. Predictors of the Z-score of changes in Well-Being across Waves

Income < $50,(	-0.018<br>(-0.059 -<br>0.023)	0.003<br>(-0.035 -<br>0.041)	-0.075<br>(-0.115 - -<br>0.036)	0.056<br>(0.017 -<br>0.095)	-0.024<br>(-0.059 -<br>0.012)	0.121<br>(0.081 -<br>0.161)
Observations	16,195	16,196	16,194	16,191	16,198	16,188
R-squared	0.007	0.001	0.019	0.005	0.004	0.016

These estimates form the basis of figure 2. All models also include a constant and an indicator missing COVID-19 death rate data. In those instances, counties were coded as having zero deaths at the time of interivew. Standard errors are clustered by county. Robust 95% confidence intervals are reported in parentheses
