# Supplementary material for: Varied and unexpected changes in the well-being of seniors in the United States amid the COVID-19 pandemic
Source: PLoS One. 2021 Jun 17;16(6):e0252962. doi: 10.1371/journal.pone.0252962 (PMC8211190; doi:10.1371/journal.pone.0252962)
Supplement: S4 Table — (PDF) [file pone.0252962.s010.pdf]

S4 Table. Predictors of Changes in Well-Being across Waves

VARIABLES	depressive symptoms	pain	negative affect	positive affect	self-rated health	Cantril ladder
Extreme death rate	0.013 (-0.003 - 0.030)	0.014 (-0.012 - 0.040)	0.127 (0.055 - 0.200)	-0.034 (-0.073 - 0.005)	0.001 (-0.033 - 0.033)	-0.045 (-0.140 - 0.051)
High chance of virus	0.01 (0.001 - 0.019)	0.007 (-0.009 - 0.023)	0.173 (0.126 - 0.221)	-0.05 (-0.074 - -0.025)	0.019 (0.000 - 0.037)	-0.135 (-0.181 - -0.088)
High chance of dying	0.017 (0.007 - 0.026)	0 (-0.016 - 0.016)	0.13 (0.085 - 0.174)	-0.035 (-0.060 - -0.011)	0.034 (0.018 - 0.051)	-0.091 (-0.138 - -0.044)
Kept from exercise	0.028 (0.018 - 0.038)	-0.015 (-0.033 - 0.002)	0.103 (0.056 - 0.150)	-0.05 (-0.076 - -0.024)	0.038 (0.020 - 0.055)	-0.086 (-0.131 - -0.040)
Lost job/income	0.027 (0.013 - 0.042)	-0.024 (-0.049 - -0.000)	0.144 (0.079 - 0.209)	-0.05 (-0.083 - -0.016)	0.004 (-0.021 - 0.029)	-0.3 (-0.380 - -0.220)
Female	0.022 (0.012 - 0.031)	0.004 (-0.012 - 0.019)	0.131 (0.087 - 0.175)	-0.021 (-0.046 - 0.004)	-0.005 (-0.023 - 0.012)	-0.092 (-0.139 - -0.045)
No college	0.006 (-0.004 - 0.017)	-0.005 (-0.022 - 0.012)	-0.038 (-0.083 - 0.006)	0.005 (-0.021 - 0.030)	0.005 (-0.013 - 0.023)	0.01 (-0.040 - 0.060)
Married	0.008 (-0.004 - 0.019)	0.016 (-0.000 - 0.033)	0.063 (0.015 - 0.112)	-0.011 (-0.039 - 0.017)	-0.001 (-0.020 - 0.019)	-0.085 (-0.145 - -0.026)
Retired at wave1	0.01 (-0.000 - 0.020)	-0.005 (-0.022 - 0.012)	0.075 (0.034 - 0.117)	-0.014 (-0.037 - 0.010)	0.028 (0.007 - 0.049)	-0.13 (-0.179 - -0.082)
Nonwhite	0.004 (-0.012 - 0.020)	0.005 (-0.019 - 0.029)	-0.063 (-0.141 - 0.016)	-0.002 (-0.047 - 0.043)	-0.007 (-0.037 - 0.022)	0.047 (-0.048 - 0.142)

S4 Table. Predictors of Changes in Well-Being across Waves

Income < $50,000 at wave 1	-0.005 (-0.017 - 0.007)	0.001 (-0.017 - 0.020)	-0.099 (-0.150 - -0.047)	0.042 (0.013 - 0.072)	-0.013 (-0.032 - 0.006)	0.182 (0.122 - 0.242)
Observations	16,195	16,196	16,194	16,191	16,198	16,188
R-squared	0.007	0.001	0.019	0.005	0.003	0.013

All models also include a constant and an indicator missing COVID-19 death rate data. In those instances, counties were coded as having zero deaths at the time of interivew. Standard errors are clustered by county. Robust 95% confidence intervals are reported in parentheses
