# Supplementary material for: Varied and unexpected changes in the well-being of seniors in the United States amid the COVID-19 pandemic
Source: PLoS One. 2021 Jun 17;16(6):e0252962. doi: 10.1371/journal.pone.0252962 (PMC8211190; doi:10.1371/journal.pone.0252962)
Supplement: S5 Table — (PDF) [file pone.0252962.s011.pdf]

S5 Table. Depressive Symptoms in Jan/Feb vs. Apr/May in the 2018 Medical Expenditure Panel Survey

	Ages 60 to 68	Ages 60 and over	Ages 55 to 68	Ages 55 and over
Jan/Feb	6.32%	8.04%	6.99%	8.08%
Apr/May	5.35%	6.55%	5.17%	6.15%
obs	1,965	4,287	3,069	5,391
difference	-0.97%	-1.48%	-1.81%	-1.93%
p-value	0.409	0.097	0.056	0.014

This table shows the prevalence of depressive symptoms based on the PHQ-2 in the 2018 Medical Expenditure Panel Survey (MEPS). Jan/Feb versus Apr/May refers to respondents interviewed in these months in the 2018 MEPS.
