# Supplementary material for: Varied and unexpected changes in the well-being of seniors in the United States amid the COVID-19 pandemic
Source: PLoS One. 2021 Jun 17;16(6):e0252962. doi: 10.1371/journal.pone.0252962 (PMC8211190; doi:10.1371/journal.pone.0252962)
Supplement: Attachment — Submitted filename: PLOS ONE.docx [file pone.0252962.s012.docx]

PLOS ONE

Varied and Unexpected Changes in the Well-Being of Seniors in the United States

amid the COIVD-19 Pandemic

The authors examine and present changes in several measures of well-being collected in 2-waves of a national, online panel survey of seniors aged 60-68, before and during the COVID-19 pandemic. In the Introduction, the authors note that prior studies of pandemic-induced changes in well-being of older adults have relied on cross-sectional data or follow respondents post-pandemic. Thus, the significance of the study derives from the panel study design, allowing assessments of well-being before versus after the start of the pandemic. Most comments below request more information/details about the study.

1. Perhaps the most important comment is that the Introduction section lacks information about the context of the pandemic in the U.S. in 2020. Contextualizing the pandemic is important because context is the basis for (or the reasons) why older adults (and other age groups) might be worse off after the pandemic, compared to before the pandemic. Salient elements of context include, but are not limited to, such as societal changes as the shutdown of the U.S. economy, stay-at-home orders, working from home (when possible), wearing masks, social distancing, closing schools, reduced access to grandchildren and aging parents in nursing homes, and so on. All of these, taken together, are compelling contextual reasons why people’s well-being might decline during the pandemic. Also, five or ten years from now, readers’ memories of the pandemic’s societal impacts may have faded; adding information about context will be a useful reminder to future readers.

Also, related to the above comment, while the panel study design is great, there is no control group: there is no geographic area of the U.S. that is not exposed to the coronavirus. Therefore, it is impossible to estimate how much well-being might have changed in the absence of the pandemic. Does the literature indicate that the secular trend for this age group is one of increasing or decreasing well-being over time, or stable well-being over time?

2. In the Introduction section on page 3, there is a sentence noting that past studies have found that seniors have fared better than other age groups during the pandemic. Why is this so?

3. In the Introduction, the authors hypothesize that changes in well-being may vary by baseline characteristics, such as gender and education. What is the basis (or reasons) for the hypothesized variation?

4. The following comments are for the Methods section:

The baseline survey runs from November 2019 to February 2020. The second wave survey began in April 2020. When did the second wave survey end?

Page 4 indicates respondents were matched to the COVID-19 death count in their county of residence at wave 2. However, in the Results section, findings are presented for COVID-19 rates rather than counts.

In the measures section, please clarify whether the pain measure is for physical pain. On the Web, the documentation for the Cantril ladder indicates the top of the ladder is defined as the best possible life, and the bottom of the ladder is defined as the worst possible life. Consider noting those anchors in the text.

In the regression models with the COVID-19 variables, did those regression models include fixed effects, like in the first set of models?

5. The following comments are for the Results section:

On page 7, the authors describe the change in negative affect off the base. Please add the base value for positive affect and for the Cantril ladder. About 40% of respondents reported pain in wave 1, which sounds high – perhaps there was regression toward the mean?

In Table 1, why are the sample sizes smaller for depressive symptoms and pain?

In Table 1, please consider adding a column on the far right reporting the differences in the means, and whether the differences are significant. Is the statistical test for differences in wave 1 versus wave 2 a bivariate test (unadjusted)? If t-tests are conducted, were paired t-tests conducted?

Table 1 just contains descriptive statistics for the dependent variables. Please add a table indicating descriptive statistics for sociodemographic characteristics in wave 1.

In the footnotes to Figure 1 and Figure 2, please add a sentence describing the regression models that produced the estimates in each Figure.

There are increasing reports in the literature that the pandemic is having more severe impacts on vulnerable groups. In Figure 1, does the data set allow comparison of low-come versus other incomes? Comparisons of other racial groups within the broad “nonwhite” category?

6. In the Discussion section, the authors note that self-rated health did not change between wave 1 and wave 2. Does this finding suggest that general measures of health status are less susceptible to change than the more specific measures of emotional well-being?
