# Supplementary material for: Varied and unexpected changes in the well-being of seniors in the United States amid the COVID-19 pandemic
Source: PLoS One. 2021 Jun 17;16(6):e0252962. doi: 10.1371/journal.pone.0252962 (PMC8211190; doi:10.1371/journal.pone.0252962)
Supplement: Attachment — Submitted filename: Response to Reviewers Final.docx [file pone.0252962.s013.docx]

PLOS ONE Response to Reviewers

Varied and Unexpected Changes in the Well-Being of Seniors in the United States amid the COVID-19 Pandemic

We thank the reviewer for the feedback. We have listed his comments below in italics and our responses in normal font.

*1. Perhaps the most important comment is that the Introduction section lacks information about the context of the pandemic in the U.S. in 2020. Contextualizing the pandemic is important because context is the basis for (or the reasons) why older adults (and other age groups) might be worse off after the pandemic, compared to before the pandemic. Salient elements of context include, but are not limited to, such as societal changes as the shutdown of the U.S. economy, stay-at-home orders, working from home (when possible), wearing masks, social distancing, closing schools, reduced access to grandchildren and aging parents in nursing homes, and so on. All of these, taken together, are compelling contextual reasons why people’s well-being might decline during the pandemic. Also, five or ten years from now, readers’ memories of the pandemic’s societal impacts may have faded; adding information about context will be a useful reminder to future readers.*

We thank the reviewer for this great point. We have added a discussion to the introduction (first paragraph) of the many ways that the pandemic affected the everyday life of older Americans.

*2. Also, related to the above comment, while the panel study design is great, there is no control group: there is no geographic area of the U.S. that is not exposed to the coronavirus. Therefore, it is impossible to estimate how much well-being might have changed in the absence of the pandemic. Does the literature indicate that the secular trend for this age group is one of increasing or decreasing well-being over time, or stable well-being over time?*

This is also a good point that we now discuss on lines 360-361. In the MEPS data, the PHQ-2 questions are asked of respondents in 2018 across the months of January and July. Looking at respondents ages 60 to 68 interviewed in January and February (respondents weren’t interviewed in November or December) versus in April and May, we see a *decline* in rates of depressive symptoms of about 1 percentage point from about 6.3% to 5.3%. The sample is relatively small (N=1,965) and the difference not statistically distinguishable from zero. But expanding the sample to include, for example, those 55 to 68 generates larger and statistically significant declines in depressive symptoms (see Supplemental Table 5). If this decline reflects normal seasonality, then our finding of an increase in depressive symptoms during the pandemic may be understated. That is, to the extent that seasonality impacts well-being, an issue that is not completely settled in the literature, depression scores should peak in the winter (Lyall et al. 2018). Since our first data collection wave happened in the winter and our second wave in the spring, this would imply that the declines in mood and affect in our data are, if anything, underestimates of the effects of the pandemic.

Likewise, the literature has documented a U-shaped pattern of global and positive hedonic well-being with age with the lowest level of average well-being around age 50 (Stone et al. 2010, Stone et al. 2020). Therefore, in our age group (60-68 year-olds) we would expect an increase in well-being (e.g., decline in depressive symptoms) over time (as respondents age), which is the opposite of what we find during the pandemic. We have added discussion of these points to the limitations section of the paper.

*3. In the Introduction section on page 3, there is a sentence noting that past studies have found that seniors have fared better than other age groups during the pandemic. Why is this so?*

This sentence was given too much attention in the prior version of the paper. We changed the paper to make it clearer that this was overemphasized. Answering your question, though, no one knows for sure, but Vahia et al. (2020) puts forth many potential reasons. Older adults tend to have lower stress reactivity, and in general, better emotional regulation and well-being than younger adults (Lee et al. 2019). Therefore, their resilience in face of the pandemic may reflect an interaction between internal factors (e.g. biological stress response, cognitive capacity) and external resources (e.g., social status, financial stability). Moreover, a number of coping mechanisms to deal with the effects of loneliness and isolation, including social connectedness and access to mental health care, seems to counter some of the effects of the pandemic (Hamm et al. 2020). The quality rather than the number of social connections may also be a factor (van Tilburg et al. 2020). Finally, wisdom – and its compassion component – is inversely correlated with loneliness and is more prevalent among older than younger adults (Jeste et al. 2020). Despite all these reasons, Vahia et al. (2020) note that it’s not clear that seniors will continue to do better relative to other age groups as the pandemic continues for a longer time period.

*4. In the Introduction, the authors hypothesize that changes in well-being may vary by baseline characteristics, such as gender and education. What is the basis (or reasons) for the hypothesized variation?*

We now discuss on lines 83-88 how the heterogeneity analysis was exploratory in nature given the uncertain effects of the pandemic but that it was motivated by the potential for COVID-19 to differentially affect individuals based on circumstances such as caregiver status, work status, and ability to work from home, and financial exposure, which are likely mediated by education, gender, race and other “pre-determined” characteristics in our survey.

*The following comments are for the Methods section:*

*5. The baseline survey runs from November 2019 to February 2020. The second wave survey began in April 2020. When did the second wave survey end?*

The second wave ended in late May 2020. We have added this information to the Methods section of the revised paper (see line 100).

*6. Page 4 indicates respondents were matched to the COVID-19 death count in their county of residence at wave 2. However, in the Results section, findings are presented for COVID-19 rates rather than counts.*

Thank you for bringing this up. We have matched participants to county-level COVID-19 death counts as well as population from the 2018 American Community Survey which we now note (see line 111). We have transformed death counts to rates – by dividing counts by county population – in order to account for the wide variation in county size. We have now clarified this in the paper (see lines 112-113).

*7. In the measures section, please clarify whether the pain measure is for physical pain. On the Web, the documentation for the Cantril ladder indicates the top of the ladder is defined as the best possible life, and the bottom of the ladder is defined as the worst possible life. Consider noting those anchors in the text.*

Yes, our pain measure refers to physical pain. The specific question is:

“Did you experience the following feelings during A LOT OF THE DAY yesterday? Yesterday, did you experience physical pain?” We now clarify this on line 136.

We have noted this in the measures section, as well as the anchors in the Cantril ladder question (which is a commonly used measure of subjective well-being). See lines 145-147.

*8. In the regression models with the COVID-19 variables, did those regression models include fixed effects, like in the first set of models?*

The regression model is in first difference, which is equivalent to including individual fixed effects. We have tried to clarify this by moving the statistical models from the appendix to the methods section. See lines 159-175 for discussion of the first difference model. We have also changed the outcome to a Z-score of the first difference to facilitate comparisons across outcomes.

*The following comments are for the Results section:*

*9. On page 7, the authors describe the change in negative affect off the base. Please add the base value for positive affect and for the Cantril ladder. About 40% of respondents reported pain in wave 1, which sounds high – perhaps there was regression toward the mean?*

We have added the base value for positive affect (1.69) and Cantril ladder (7.30) to the text in lines 211-216, also listed in Table 1. We also discuss estimates on pain that were recently published by one of us (A.A.S). See lines 193-195.

*10. In Table 1, why are the sample sizes smaller for depressive symptoms and pain?*

We apologize for the confusion. We were listing the size of the affected group (i.e. the number of people with depressive symptoms/reporting pain). We have now changed it to the per wave sample size for each variable, so that the slight variation in sample sizes now reflects missing responses. Note that we require respondents answer in both waves to be in our analysis for that variable so the sample size is the same across each wave.

*11. In Table 1, please consider adding a column on the far right reporting the differences in the means, and whether the differences are significant. Is the statistical test for differences in wave 1 versus wave 2 a bivariate test (unadjusted)? If t-tests are conducted, were paired t-tests conducted?*

Thanks. We have revamped Table 1 (now Table 2). In addition to sample size/or number of respondents, it now has a column that shows the difference across waves and the p-value from a paired t-test of the difference in means.

*12. Table 1 just contains descriptive statistics for the dependent variables. Please add a table indicating descriptive statistics for sociodemographic characteristics in wave 1.*

Thanks for this comment. This information was in Supplemental Table 2 in our original submission. We have now made this Table 1.

*13. In the footnotes to Figure 1 and Figure 2, please add a sentence describing the regression models that produced the estimates in each Figure.*

Thanks for these comments. We have added the estimating equations to the main text, instead of relegating them to the appendix, and now refer to them in the figure footnotes.

*14. There are increasing reports in the literature that the pandemic is having more severe impacts on vulnerable groups. In Figure 1, does the data set allow comparison of low-come versus other incomes? Comparisons of other racial groups within the broad “nonwhite” category?*

We have added to Figures 1 and 2 analysis for income below $50,000 versus above and including $50,000 and discuss them in the main text. Surprisingly, we find that the worsening of depressive symptoms and well-being across waves was larger among the high-income respondents than the low-income respondents. As mentioned in the revised paper (see lines 338-340), the reasons for this are unclear and potentially complex, a subject we have been exploring in ongoing work. Unfortunately, as indicated by the large confidence intervals on the “non-white” variable in Figures 1 and 2, we do not have enough sample of different racial groups to run analyses further dividing by racial groups.

*15. In the Discussion section, the authors note that self-rated health did not change between wave 1 and wave 2. Does this finding suggest that general measures of health status are less susceptible to change than the more specific measures of emotional well-being?*

Possibly. When health changes, well-being changes but the reverse is not necessarily true. While we did find the results surprising, we hesitate to generalize based on our results.

References:

Hamm, M.E., Brown, P.J., Karp, J.F., Lenard, E., Cameron, F., Dawdani, A., Lavretsky, H., Miller, J.P., Mulsant, B.H., Pham, V.T. and Reynolds, C.F., 2020. Experiences of American older adults with pre-existing depression during the beginnings of the COVID-19 pandemic: a multicity, mixed-methods study. *The American journal of geriatric psychiatry*, *28*(9), pp.924-932.

Jeste, D.V., Di Somma, S., Lee, E.E., Nguyen, T.T., Scalcione, M., Biaggi, A., Daly, R., Liu, J., Tu, X., Ziedonis, D. and Glorioso, D., 2020. Study of loneliness and wisdom in 482 middle-aged and oldest-old adults: a comparison between people in Cilento, Italy and San Diego, USA. *Aging & Mental Health*, pp.1-11.

Lee, E.E., Depp, C., Palmer, B.W., Glorioso, D., Daly, R., Liu, J., Tu, X.M., Kim, H.C., Tarr, P., Yamada, Y. and Jeste, D.V., 2019. High prevalence and adverse health effects of loneliness in community-dwelling adults across the lifespan: role of wisdom as a protective factor. *International psychogeriatrics*, *31*(10), p.1447.

Lyall, L.M., Wyse, C.A., Celis-Morales, C.A., Lyall, D.M., Cullen, B., Mackay, D., Ward, J., Graham, N., Strawbridge, R.J., Gill, J.M. and Ferguson, A., 2018. Seasonality of depressive symptoms in women but not in men: A cross-sectional study in the UK Biobank cohort. *Journal of affective disorders*, *229*, pp.296-305.

Stone, A.A., Schwartz, J.E., Broderick, J.E. and Deaton, A., 2010. A snapshot of the age distribution of psychological well-being in the United States. *Proceedings of the National Academy of sciences*, *107*(22), pp.9985-9990.

Stone, A.A., Broderick, J.E., Wang, D. and Schneider, S., 2020. Age patterns in subjective well-being are partially accounted for by psychological and social factors associated with aging. *Plos one*, *15*(12), p.e0242664.

Vahia, I.V., Jeste, D.V. and Reynolds, C.F., 2020. Older Adults and the Mental Health Effects of COVID-19. *JAMA*, *324*(22), pp.2253-2254.

Van Tilburg, T.G., Steinmetz, S., Stolte, E., van der Roest, H. and de Vries, D.H., 2020. Loneliness and mental health during the COVID-19 pandemic: A study among Dutch older adults. *The Journals of Gerontology: Series B*.

Answer to Journal Requirements:

1. *Please ensure that your manuscript meets PLOS ONE's style requirements, including those for file naming.*

Thanks. We have tried to ensure we meet the *PLOS ONE's style requirements.*

2. *Please provide additional details regarding participant consent. In the ethics statement in the Methods and online submission information, please ensure that you have specified (1) whether consent was informed and (2) what type you obtained.*

Participants were shown a consent form online before they answered the online survey. They gave us consent by clicking “next” and entering the survey. We have updated the Methods section with this information and also added the consent form to the Supporting Information file (S5 File).

3. *In the Methods section and the online submission, please further clarification whether the original study, conducted between November 2019 and February 2020 received ethical approval and please provide the ethical approval number.*

Yes, it did. The USC IRB approval number is UP-20-00259. We clarify this on lines 115-117.

4. *Please include additional information regarding the survey or questionnaire used in the study and ensure that you have provided sufficient details that others could replicate the analyses. For instance, if you developed a questionnaire as part of this study and it is not under a copyright more restrictive than CC-BY, please include a copy, in both the original language and English, as Supporting Information.*

We have added both our survey questionnaires to the Supporting Information (S1 File and S2 File).

5. *In your Data Availability statement, you have not specified where the minimal data set underlying the results described in your manuscript can be found. PLOS defines a study's minimal data set as the underlying data used to reach the conclusions drawn in the manuscript and any additional data required to replicate the reported study findings in their entirety. All PLOS journals require that the minimal data set be made fully available.*

We have included as supporting information a minimal analytic dataset. They are provided as S3. File.

6. *We note that the grant information you provided in the ‘Funding Information’ and ‘Financial Disclosure’ sections do not match. When you resubmit, please ensure that you provide the correct grant numbers for the awards you received for your study in the ‘Funding Information’ section.*

The funding information is now complete. It does require adjusting the *Financial Disclosure section to list:*

NIA K01AG050811-03S1 – Silvia Barcellos

NIA K01AG050811-05S1 - Silvia Barcellos

Navigage Foundation – Mireille Jacobson

7. *Please include your tables as part of your main manuscript and remove the individual files. Please note that supplementary tables (should remain/ be uploaded) as separate "supporting information" files.*

Main tables 1 and 2 are now in the main text.

8. *Please include captions for your Supporting Information files at the end of your manuscript, and update any in-text citations to match accordingly. Please see our Supporting Information guidelines for more information:* [*http://journals.plos.org/plosone/s/supporting-information*](http://journals.plos.org/plosone/s/supporting-information)*.*

Supplemental Tables 1-5 are now listed at the end of our main document and will be uploaded as separate files.
