# Supplementary material for: Varied and unexpected changes in the well-being of seniors in the United States amid the COVID-19 pandemic
Source: PLoS One. 2021 Jun 17;16(6):e0252962. doi: 10.1371/journal.pone.0252962 (PMC8211190; doi:10.1371/journal.pone.0252962)
Supplement: Attachment — Submitted filename: PLOS ONE revision.docx [file pone.0252962.s014.docx]

PLOS ONE

Varied and Unexpected Changes in the Well-Being of Seniors in the United States

amid the COIVD-19 Pandemic

Revision

The authors examine and present changes in several measures of well-being collected in 2-waves of a national, online panel survey of seniors aged 60-68, before and during the COVID-19 pandemic. The revised manuscript is responsive to comments raised in the prior original manuscript. Some minor comments are presented below for consideration.

1. The Introduction section is very engaging and should be well-received by readers. A minor point is that, in the last sentence of the Introduction, the authors note that COVID-19 may have differential effects on the study’s dependent variables, which are likely mediated by education, gender, race and other pre-determined characteristics. This sentence may trigger impressions, in the minds of readers, that the authors will be doing a mediation analysis, which is not the case. Some options might be to change this sentence, or perhaps retain the sentence, as is, but touch on this “mediation theme” in the Discussion section.

2. In the Measures section, for clarity, please note the range of the negative affect scale and the range of the positive affect scale.

3. For clarity, in the Statistical Analysis section, please add a sentence defining the terms in Equation 1.

4. The Results section begins with comparisons of the study’s survey characteristics with national representative data sets. These comparisons are great. However, the Methods section does not indicate that such comparisons will be performed.

5. In Table 1, the authors and editor might consider simply noting the sample sizes a single time at the top of the columns. But please note the 2901 MEPS sample size for depression in the (b) footnote.

6. Could the decline in pain be regression to the mean?

7. Some of the dependent variables unexpectedly declined more for people with incomes above $50,000. Could this be because the pandemic led to a shutdown in the economy and increased unemployment and/or reduced hours worked per week, and people in the above $50,000 category had “more to lose” than people who had low incomes pre-pandemic – resulting in greater declines in the Cantril ladder well-being scores for the higher income group (?).
