# Supplementary material for: Varied and unexpected changes in the well-being of seniors in the United States amid the COVID-19 pandemic
Source: PLoS One. 2021 Jun 17;16(6):e0252962. doi: 10.1371/journal.pone.0252962 (PMC8211190; doi:10.1371/journal.pone.0252962)
Supplement: Attachment — Submitted filename: Response to Reviewer R2.docx [file pone.0252962.s015.docx]

PLOS ONE Response to Reviewers

Varied and Unexpected Changes in the Well-Being of Seniors in the United States amid the COVID-19 Pandemic

We thank the reviewer for the feedback. We have listed his comments below in italics and our responses in normal font.

1. *The Introduction section is very engaging and should be well-received by readers. A minor point is that, in the last sentence of the Introduction, the authors note that COVID-19 may have differential effects on the study’s dependent variables, which are likely mediated by education, gender, race and other pre-determined characteristics. This sentence may trigger impressions, in the minds of readers, that the authors will be doing a mediation analysis, which is not the case. Some options might be to change this sentence, or perhaps retain the sentence, as is, but touch on this “mediation theme” in the Discussion section.*

Thanks for this point. We now no longer use the term “mediate’ but instead say “related to” on line 88.

2. *In the Measures section, for clarity, please note the range of the negative affect scale and the range of the positive affect scale.*

Thanks. We note the scales on lines 142-3.

3. *For clarity, in the Statistical Analysis section, please add a sentence defining the terms in Equation 1.*

Sorry for this oversight. We now define terms. See lines 163-166.

4. *The Results section begins with comparisons of the study’s survey characteristics with national representative data sets. These comparisons are great. However, the Methods section does not indicate that such comparisons will be performed.*

We now describe this in the Methods section. See lines 154-158.

5. *In Table 1, the authors and editor might consider simply noting the sample sizes a single time at the top of the columns. But please note the 2901 MEPS sample size for depression in the (b) footnote.*

This is a great suggestion. We have changed the table as recommended.

6. *Could the decline in pain be regression to the mean?*

It certainly could be but with two data points we cannot really say much about this here. Further work, where we analyze subsequent data waves, might be able to share light on the role of regression to the mean. Here we simply document these changes and note that the decline in pain goes in the “opposite” direction of the large increases in depressive symptoms and negative mood.

7. *Some of the dependent variables unexpectedly declined more for people with incomes above $50,000. Could this be because the pandemic led to a shutdown in the economy and increased unemployment and/or reduced hours worked per week, and people in the above $50,000 category had “more to lose” than people who had low incomes pre-pandemic – resulting in greater declines in the Cantril ladder well-being scores for the higher income group (?).*

This is certainly a possibility. Other possible explanations are the steep decline in the value of stocks during this time (which likely disproportionally affected the high-income group) and the government pandemic cash assistance (which might have mitigated income loss and well-being reductions for the low-income group). We are currently investigating what might be behind this unexpected result in other work.
